# Editorial: Tumor Vessels as Directors of the Tumor Microenvironment: New Findings, Current Challenges & Perspectives

**DOI:** 10.3389/fcell.2022.885670

**Published:** 2022-03-29

**Authors:** Lucas Treps, Ann Ager, Kyoko Hida

**Affiliations:** ^1^ Nantes Université, Inserm UMR 1307, CNRS UMR 6075, Université d’Angers, CRCI2NA, Nantes, France; ^2^ Division of Infection and Immunity, School of Medicine, Cardiff University, Cardiff, United Kingdom; ^3^ Vascular Biology and Molecular Biology, Graduate School of Dental Medicine, Hokkaido University, Sapporo, Japan

**Keywords:** tumor endothelial cell, angiogenesis, tumor microenvironment, extracellular vesicles, anti-tumor immunity, immunotherapy, anti-angiogenic therapies

## Introduction

Half a century ago, Judah Folkman postulated that solid tumors rely on the formation of new blood vessels (angiogenesis) from pre-existing established vessels for appropriate nutrient and oxygen supply (Folkman, 1971). Accordingly, tumor spheroids infiltrated with new capillaries would exponentially grow, while avascular tumors remain dormant. This original premise was the cornerstone for the development of anti-angiogenic therapies, aiming at starving and asphyxiating the tumor to death by destroying tumor blood vessels. Since that time, intense research has been conducted to identify angiogenic factors controlling tumor angiogenesis. Several pro-angiogenic cues were discovered, including the vascular endothelial growth factor (VEGF) first characterized by the Dvorak laboratory (Senger et al., 1983). VEGF is probably one of the most notable angiogenic factors, and in the late 90’s, nearly a decade after its discovery, a humanized monoclonal neutralizing antibody was developed by the team of Napoleone Ferrara which shows promising anti-tumor effects (Presta et al., 1997). This was a real breakthrough and paved the way for the development of numerous anti-angiogenic strategies that are now approved in clinics for eye diseases (i.e., wet age-related macular degeneration) and anti-cancer treatments. Even though such therapies improved patients’ survival, they are still facing resistance mechanisms that partly results from our incomplete understanding of the molecular mechanisms and signaling pathways that govern tumor angiogenesis. Additionally, far from being incapable of genetic plasticity (as initially suggested by Folkman) tumor endothelial cells are heterogeneous by essence and harbor a plethora of phenotypes with varying sensitivity to anti-angiogenic therapies (Goveia et al., 2020). This complexity in tumor vessels also implies a richer variety of cell-cell interactions within the tumor microenvironment than anticipated.

**FIGURE 1:**
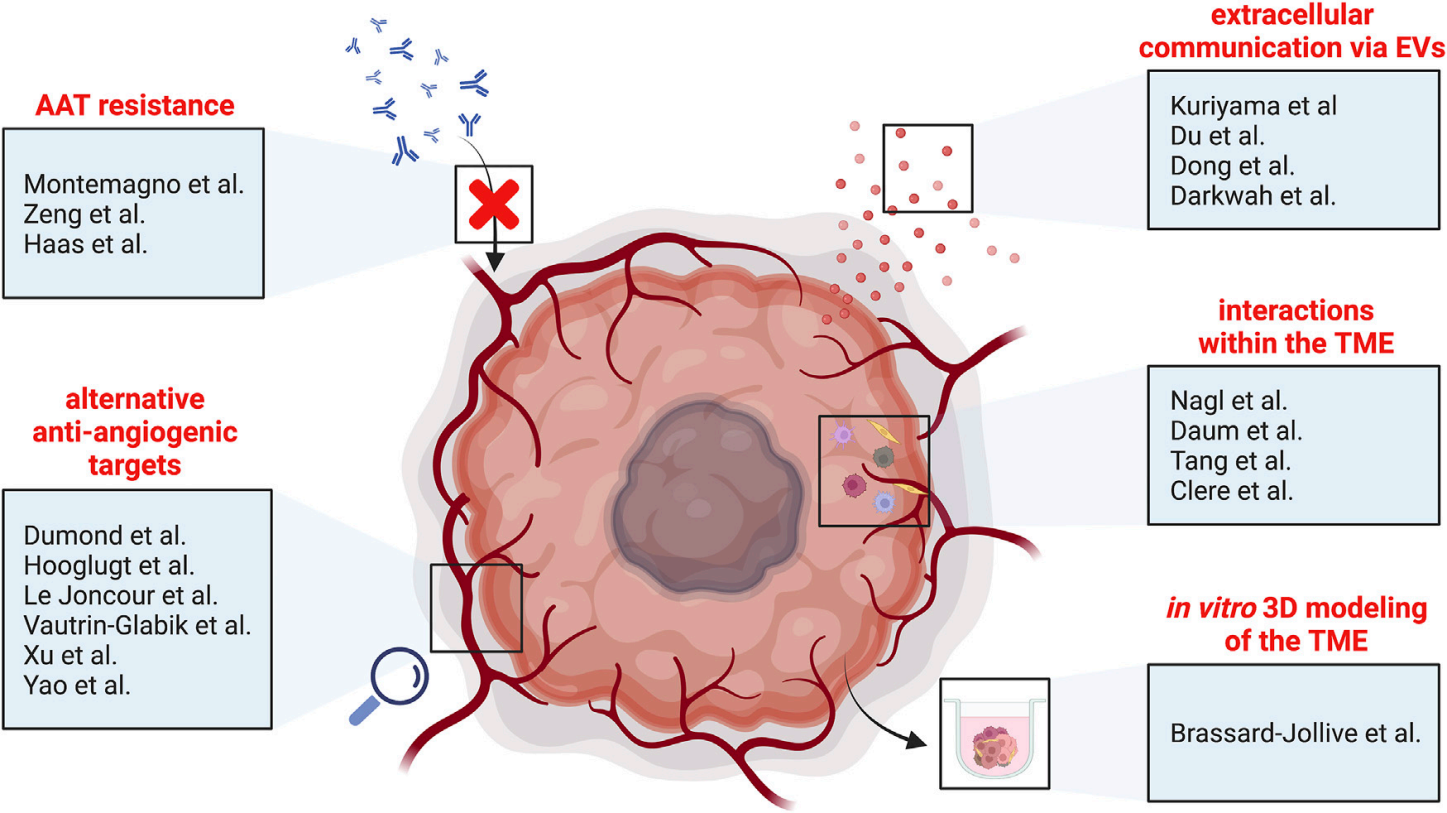
Schematic overview of the different topics covered by this special research topic of “*Tumor Vessels as Directors of the Tumor Microenvironment: New Findings, Current Challenges & Perspectives*”. AAT, anti-angiogenic therapy; EVs, extracellular vesicles; TME, tumor microenvironment.

This special research topic of “*Tumor Vessels as Directors of the Tumor Microenvironment: New Findings, Current Challenges & Perspectives*” comprises seven original research articles and eleven review articles for a total of 18 original contributions. These articles cover many topics articulated around tumor vessels and scrutinize mechanisms of resistance to anti-angiogenic therapies, molecular signaling driving and regulating pathological angiogenesis, and recent approaches modulating tumor vessels and their micro-environment (Figure 1). We will discuss these aspects in this editorial and hope to provide insights into the complexity of tumor vessel biology.

## The Widespread Dissimilarities Between Normal and Tumor Endothelial Cells

A striking difference between normal and tumor vessels is morphology - the former exhibit a smooth lining of endothelial cells (ECs) that are mature and hierarchically organized, while the latter show irregular and tortuous endothelium, with higher permeability and impaired perfusion (Figure 1). In addition, it has been reported that tumor endothelial cells (TECs) are different from normal endothelial cells (NECs) in many aspects such as morphology, molecular, transcriptomic, metabolic profiles, and cytogenetically. Technological breakthroughs from the last decade with single cell transcriptomics (scRNA-seq), has unveiled the wide diversity of cellular and molecular composition of the tumor microenvironment (TME). As such, recent studies have shown that TECs are composed of heterogeneous populations/phenotypes and adapt their traits in response to the TME (e.g., hypoxia or reactive oxygen species). Also, surrounding tumor cells, cancer-associated fibroblasts (CAFs) and immune cells (to name a few) affect TECs. Vice versa, TEC may regulate and shape the TME by releasing angiocrine factors (Maishi et al., 2019). TECs foster tumor progression and metastasis, and even induce therapy resistance by alteration of their molecular signature during tumor progression or anti-cancer treatments (anti-angiogenic, immunotherapy). Nagl et al. provide an overview of the studies on TECs and their interactions within the TME. They focus on the role of TECs as immune regulators within their cellular habitat. Indeed, TECs can affect: 1) immune cell migration and priming, 2) T cell activation and apoptosis (e.g., by upregulating FAS-L), and 3) antigen presentation. For instance, ECs in non-cancerous tissue can express major histocompatibility complex (MHC) class I and II molecules, enabling them to present processed antigens to T cells. However, TECs downregulate genes that are responsible for MHC expression and show suppressed antigen-presenting functions (Goveia et al., 2020; Lambrechts et al., 2018; Li et al., 2021). Thus, TECs may contribute to tumor immune evasion and may influence responses to immunotherapies.

## Resistance to Anti-Angiogenic Therapies

As a spearhead into the therapeutic armory, bevacizumab was the first anti-angiogenic treatment (AAT) approved for metastatic colorectal cancer as a first line treatment combined with chemotherapy (Hurwitz et al., 2004). Thereafter, an armamentarium of molecules was developed to target pro-angiogenic cues (e.g., VEGF, FGF, PDGF) and their cognate receptors. Although some tumors display sensitivity to AATs, several cancer entities remain resilient to such treatments and demonstrated limited impact on overall survival. Intrinsic and acquired resistance are deemed to be responsible for such failures in therapeutic response. Montemagno et al. provide an interesting update on AAT resistance, by distinguishing early-stage resistance, mostly involving tumor cells with, for example, the upregulation of genes involved in angiogenic redundancy, to late-stage resistance related to the adaptation of the tumor microenvironment. In this regard, Daum et al. put forward a comprehensive overview about how highly adaptive the tumor microenvironment can be in non-small cell lung cancer. Along with these new insights, they present combinational approaches including chemotherapy, anti-angiogenic and immunotherapy which could be developed to yield a more target-oriented anti-tumor treatment. Once the tumor has disseminated throughout the body, micro-metastases can remain dormant and thus unresponsive to anti-angiogenic therapy. In their mini review article, Zeng et al. provide a concise overview of the resistance mechanisms developed by the tumor to counteract AAT. Haas et al. review another means by which micro-metastases stay beyond the reach of these drugs, namely vessel co-option whereby cancer cells hijack pre-existing abundant vasculature of the host organ (e.g., liver, lung) without the need for angiogenesis. Unexpectedly, recent pre-clinical evidence highlights the fact that co-opted vessels share transcriptomic similarities with quiescent ECs in healthy vessels and are nearly devoid of angiogenic tip and proliferating ECs, thus potentially explaining unresponsiveness to AAT (Teuwen et al., 2021).

## Alternative Approaches and New Targets to Attack Tumor Vessels

In 2001, Rakesh Jain hypothesized that AATs could be used to normalize, rather than prune and destroy tumor blood vessels (Jain, 2001). The rationale behind this paradigm was to heal tumor blood vessels, in order to improve oxygenation for successful tumor treatment and thereby: 1) breaking the vicious cycle of hypoxia-mediated tumor progression; 2) improving drug delivery, radiation- and immunotherapy efficacy (Fukumura et al., 2018); and 3) recruiting more immune cells to the tumor. One way to achieve tumor vessel normalization is by fine-tuning the dose and timing of anti-VEGF/R AATs during the window of normalization. Other promising targets include the Neuropilins, which act as receptors for the semaphorins but also as VEGF co-receptors, and are important for EC function (Treps et al., 2013), to modulate tumor permeability (Treps et al., 2016) and vessel normalization (Casazza et al., 2011). Dumond et al. overview the function of Neuropilins and their multifaceted contributions to the TME, and highlight their relevance in anti-tumor targeting. Among others, YAP/TAZ are critical regulators of developmental angiogenesis. Hooglugt et al. present an appealing review on the role of YAP/TAZ signaling as another pathway amenable to induce tumor vessel formation and cancer cell growth via oncogenic activation. In that regard, the STAT3/YAP/TAZ signaling was recently described to be critical in promoting tumor vascularization in human colorectal carcinomas and skin melanoma (Shen et al., 2021).

A better comprehension of the mechanisms driving angiogenesis is a pre-requisite to improve AATs. As such, this research topic contributed to this never-ending goal with Le Joncour et al. who investigated the role of the vasoactive peptide urotensin II and its receptor urotensin on glioblastoma angiogenesis. Interestingly, in preclinical models of glioblastoma, urotensin antagonist and biased urotensin ligand were able to significantly delay tumor growth, and evoke strong angiogenic activity through targeting integrin activation. Vautrin-Glabik et al. demonstrated the anti-angiogenic activity of a 13 amino acid sequence (dubbed QS-13) derived from the type IV collagen, presumably through its binding to α5β1 integrin onto the surface of ECs. Long non-coding RNA (LncRNA) can function as a sponge to counteract endogenous RNA including micro-RNA (miR). As such, Xu et al. described the function of the LncRNA NEAT1 in the inhibition of the miR-17-5p/TGFβR2 axis in gastric cancer lines, thereby leading to the secretion of pro-angiogenic factors in the milieu. Yao et al. showed that miR-9-induced angiogenesis occurred via targeting the sphingosine-1-phosphate receptor.

## Tumor Vessels and Their Cellular Microenvironment

Tumor vessels reside within a complex mixture of immune cells, fibroblasts and matrix molecules which together constitute the TME. The exact composition and structural organization of the TME depends on type and stage of cancer and is regulated either directly or indirectly by TECs. The recent breakthrough in cancer therapy using immune checkpoint blockade inhibitors has revealed the dominant role of immune escape in supporting cancer progression. Moreover, the success of immunotherapy has been linked to a pre-existing CD8^+^ T-cell rich and/or Foxp3^+^ Treg poor TME as well as the presence of tertiary lymphoid structures (Bruni et al., 2020). As mentioned above, TECs regulate the recruitment of distinct immune cell subsets and therefore shape the immune cell signature of the TME. Chemokine synthesis by tumor cells will also control immune cell infiltration of the TME. In this collection Tang et al. reveal a role for the transcription factor Twist1 in regulating CCL2 generation by colorectal cancer cells in the recruitment of macrophages. Interestingly, the transcription factor Forkhead Box Q1 (FOXQ1) controlled Twist1 expression by colorectal cancer cells. FOXQ1 expression by colorectal cancer cells also controlled the balance of angiogenic and angiostatic factors generated thereby directly impacting TECs in the TME.

CAFs are a heterogeneous, poorly understood cell population in the TME with a range of pro-tumoral functions. Evidence that CAFs can be generated from TECs and the signaling pathways involved in this endothelial-to-mesenchymal transition (EndMT) are reviewed by Clere et al. Interestingly, EndMT is accompanied by the development of multiple therapeutic resistance mechanisms and other pro-tumoral functions such as cancer cell extravasation and sprouting angiogenesis. Studying angiogenesis, vasculogenesis, EndMT and the functional properties of tumor blood vessels in the complexity of the TME is very challenging. Brassard-Jollive et al. comprehensively review the limitations of established 3D *in vitro* models of tube formation. New approaches using EC grown on beads and co-cultured with defined components of the TME such as tumor cells and fibroblasts within 3D hydrogels containing defined ECM components are described. However, significant challenges remain to develop 3D models of tumor blood vessels within a TME for high throughput screening required for drug testing. Another limitation is the use of non-tumor sources of EC due to the difficulties of working with isolated TECs, which could potentially preclude clinical translation.

## Tumor Vessels Extracellular Communication

Since their discovery 2 decades ago, the generic term of extracellular vesicles (EVs) has been acknowledged and encompasses a large variety of appellations such as exosomes, microvesicles, microparticles, etc. Driven by a vibrant research community (International Society for Extracellular Vesicles, ISEV), it is now well established that EVs are involved at various steps of tumor development, cell-cell communication with tumor microenvironment, and anti-cancer therapy resistance. Kuriyama et al. present a comprehensive review on the involvement of EVs in tumor vascular-related cancer progression and their contribution to AAT resistance mediated by vasculogenic mimicry. Vasculogenic mimicry is a process whereby aggressive cancer cells form *de novo* vascular networks that are associated with malignant phenotype (Treps et al., 2021). Du et al. analyzed the expression of 5-methylcytosine regulators and DNA methylation-driven genes in EVs and tissue samples in order to identify a risk signature for colon cancer. Interestingly, Dong et al. identified by micro-array analysis of EVs miR-3682-3p as repressed in highly metastatic hepatocellular carcinoma cells and shown to impair angiogenesis by targeting ANGPT1. It is worth mentioning that EVs should not always be presented as the villains during cancer. Indeed, Darkwah et al. presented muscle derived-EVs and point to their potentially protective roles in cancer progression and metastasis.

## Summary and Prospective

Initially uncovered upon characterization of ECs from different vascular beds, endothelial heterogeneity is now acknowledged in a broad variety of pathologies including cancers, whereby various phenotypes of TECs have a myriad of possible cell-cell interactions. The complexity and dynamics of these interplays are challenging to study in the patient either using resections or biopsies. However, the advent of scRNA-seq, multiplexed imaging, spatially resolved sequencing technologies and computational strategies inferring cell–cell interactions have opened novel avenues in deciphering communications occurring within the TME.

The immunological properties of ECs is a topic under intense investigations and may offer enthralling discoveries that could be used to tailor existing anti-cancer treatments. For example, a recent pre-clinical study has shown that the secreted leucine-rich α-2-glycoprotein 1 (LRG1) is induced in TECs from lung and melanoma cancer mouse models, but also in particular human biopsies, and that LRG1 function-blocking antibody treatment results in tumor vessel normalization. Moreover, LRG1-blocking antibody combined with cisplatin chemotherapy, adoptive T cell therapy, or anti-PD1 immune checkpoint inhibition monotherapy could enhance the efficacy of these therapies (O’Connor et al., 2021). Seemingly, combination approaches using AAT and anti-PD-L1 therapy showed improved efficiency by counteracting therapy-induced adaptive immunosuppressive pathways, by enhancing the tumor infiltration of T cell via promoting high endothelial venule (HEV) formation (Allen et al., 2017). Indeed, tumor-associated HEVs represent the main site of lymphocytes extravasation upon combined anti-PD-1/anti-CTLA-4 immunotherapy, and it was recently uncovered that LTßR-mediated HEV maturation is crucial to improve the efficacy of these immunotherapies (Asrir et al., 2022). Different targeted approaches meant to foster tumor HEV generation led to promising reinforced efficacy of combination therapy in several cancer entities including pancreatic neuroendocrine tumors (Johansson-Percival et al., 2017), glioblastoma (He et al., 2018), lung metastases (He et al., 2020) but would now require clinical translation. Although, the heterogeneity of HEV from adult mouse peripheral lymph nodes has been characterized in homeostasis and inflammation (Veerman et al., 2019), the tumor HEV heterogeneity calls for in depth scrutiny to better understand how these structures are formed, and how they could be reliably induced to fight anti-tumor immunosuppression and therapy resistance mechanisms.
